# Influence of the Dickkopf-related protein 1-(co)receptor axis on early bone colonization in a subcutaneous human prostate cancer xenograft model

**DOI:** 10.1016/j.jbo.2026.100814

**Published:** 2026-10-30

**Authors:** By Marie-Therese Haider, Vera Freytag, Anke Baranowsky, Farbod Bahreini, Christof Niehrs, Thorsten Schinke, Michael Amling, Tobias Lange

**Affiliations:** a Institute of Anatomy, University of Luebeck, Luebeck, Germany; b Institute of Anatomy and Experimental Morphology, University Medical Center Hamburg-Eppendorf, Hamburg, Germany; c Department of Trauma and Orthopedic Surgery, University Medical Center Hamburg-Eppendorf, Hamburg, Germany; d Institute of Molecular Biology (IMB), Mainz, Germany; e Division of Molecular Embryology, DKFZ-ZMBH Alliance, Heidelberg, Germany; f Department of Osteology and Biomechanics, University Medical Center Hamburg-Eppendorf, Hamburg, Germany

**Keywords:** Prostate cancer, Bone metastasis, DKK1, Kremen 2, Wnt signaling

## Abstract

**Background:**

The Wnt signaling pathway drives tumor cell invasion, metastasis, and bone formation, making it a key mediator of prostate cancer (PCa) skeletal metastasis. Dickkopf-related protein 1 (DKK1) inhibits canonical Wnt signaling by binding to the Wnt co-receptor LRP5/6; the DKK1-binding receptors Kremen1/2 cooperate in this inhibition. We investigated whether targeting this axis affects early bone colonization in a subcutaneous PCa xenograft model, an approach that reflects spontaneous metastatic dissemination but is limited by low metastatic frequency and the requirement for immunodeficient hosts.

**Methods:**

Luc2/RGB-labelled PC-3 cells were implanted subcutaneously into male immunodeficient mice. DKK1 was neutralized with the humanized anti-DKK1 monoclonal antibody hDKN-01 during primary tumor growth (pre-OP) or following primary tumor surgical resection (post-OP). Further experiments used host mice with heterozygous Lrp5 gain-of-function mutation (*Lrp5*^+/G170V^) or knockout of Kremen1 (*Krm1*^−/−^), Kremen2 (*Krm2*^−/−^), or both. Bone metastatic burden was quantified using anti-luciferase immunohistochemistry on serial hindlimb sections. Serum DKK1 ELISA and bone histomorphometry confirmed treatment efficacy and genotype phenotypes.

**Results:**

hDKN-01 reduced serum DKK1 and increased trabecular bone volume, confirming biological activity, but did not affect tumor growth or bone metastatic burden in either treatment window. *Lrp5*^+/G170V^ and *Krm1*/*Krm2* double-KO mice showed expected bone phenotypes without altered bone colonization (detected in 13–33% of mice). Notably, *Krm2*^−/−^ mice showed only single disseminated tumor cells in bone, with no outgrowing colonies (0% of mice).

**Conclusions:**

Neither systemic neutralization of DKK1 nor *Lrp5* mutation nor *Krm1* or *Krm1*/*Krm2* knockout had any effect on bone colonization of subcutaneous PC-3 xenografts in immunodeficient mice. In contrast, *Krm2* knockout prevented the formation of colonizing bone metastases, apparently depending on the presence of *Krm1*.

## Highlights

• hDKN-01 lowers serum DKK1 but does not inhibit bone metastasis in NSG mice.
• Lrp5+/G170V increases bone mass without affecting metastatic burden.
• Krm2 loss blocks outgrowth of disseminated tumor cells into bone metastases.
• Concurrent Krm1 loss reverses the antimetastatic phenotype of Krm2 deficiency.

## Graphical abstract

**Figure:**
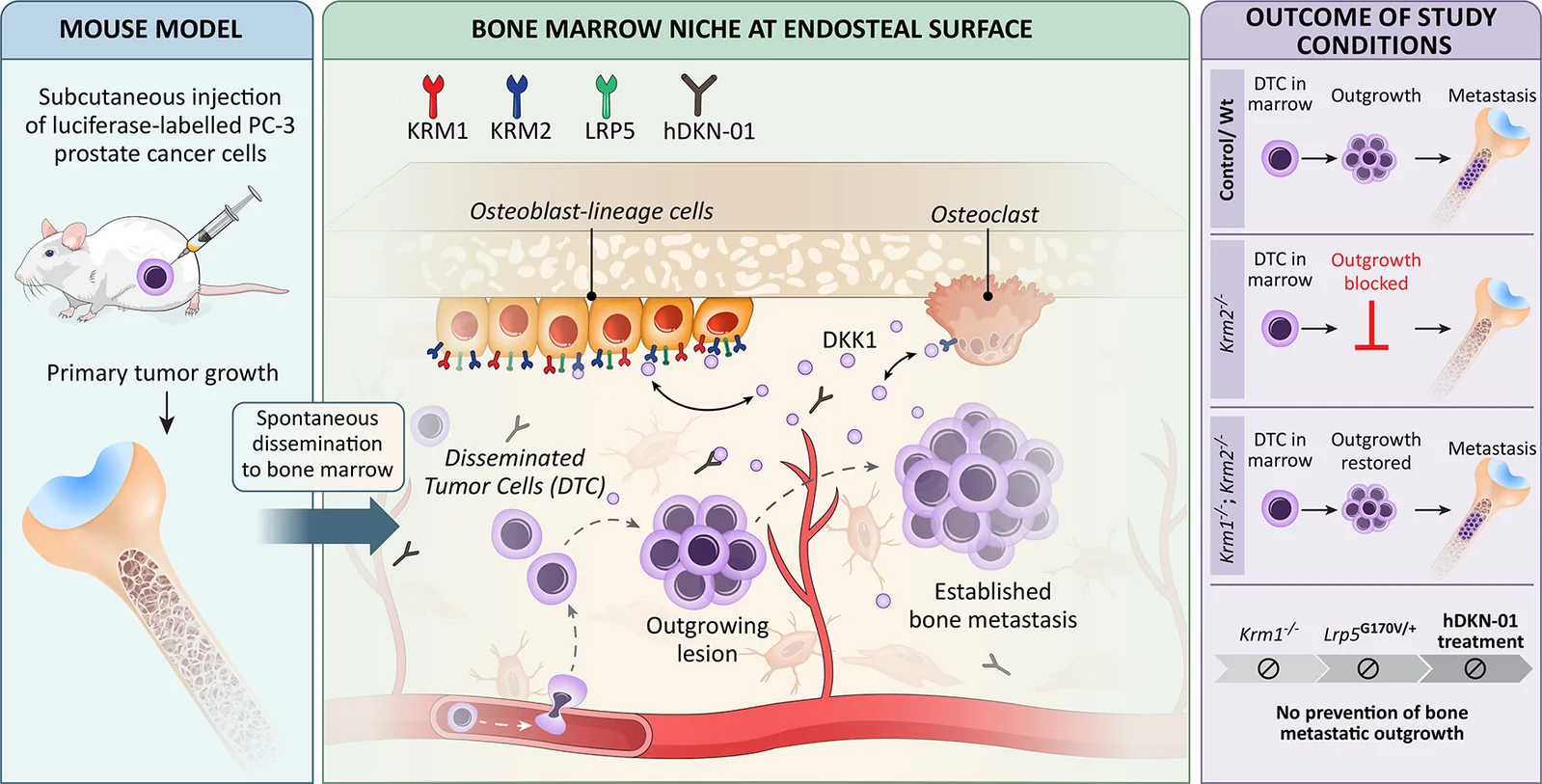

## 1 Introduction

Bone metastasis significantly contributes to morbidity and worsens prognosis in cancer patients. Prostate cancer (PCa), the second most frequently diagnosed cancer in men worldwide [1], has a particular propensity to metastasize to the skeleton [2]. Although advances in early detection and treatment have increased survival rates for localized PCa, the prognosis remains poor once the cancer becomes metastatic [3]. The molecular mechanisms driving PCa bone metastasis, however, remain incompletely understood.

PCa bone metastasis is a multistep process comprising invasion and escape from the primary tumor, entry into and survival in the circulation, bone marrow seeding, and persistence as disseminated tumor cells (DTCs). DTCs may persist without progressing to established metastatic lesions; their detection alone therefore does not establish metastatic outgrowth [4], [5]. Mouse models are required to investigate these complex processes within the interacting tumor, host, and bone microenvironment compartments.Spontaneous metastasis xenograft models retain primary tumor growth and multiple early steps of the metastatic cascade, enabling investigation of tumor- and host-dependent mechanisms of systemic dissemination and bone marrow colonization. In contrast, intracardiac and intracaudal arterial injection bypass primary tumor invasion and intravasation; intraosseous injection additionally bypasses vascular delivery and extravasation [6], [7]. Spontaneous skeletal metastasis remains difficult to model *in vivo*, with low metastatic frequency and practical limitations, including the requirement for immunodeficient hosts and - given the usually small size of metastatic lesions - limited representation of the osteoblastic phenotype of advanced human PCa bone metastases [6]. Despite these limitations, our group investigates the mechanisms of bone metastasis after subcutaneous implantation of human PCa cells into immunodeficient mice (spontaneous metastasis xenograft models) [8], [9], [10], [11], [12], [13], as this approach uniquely preserves the early steps of spontaneous metastatic dissemination.

The Wnt signaling pathway plays a pivotal role during embryonic development, adult tissue homeostasis as well as regulation of bone cell function [14], [15], [16], [17]. In cancer, aberrant activation of Wnt signaling promotes tumor cell proliferation, survival, epithelial-to-mesenchymal transition (EMT), invasion, and metastatic dissemination, and is associated with poor prognosis across multiple tumor entities [18], [19]. In PCa specifically, aberrant Wnt activation has been implicated in disease development and progression [20], [21], [22], [23]. In the bone microenvironment, however, Wnt signaling serves an opposing, osteoanabolic function: it drives osteoblast differentiation and bone formation, thereby contributing to the osteoblastic phenotype characteristic of advanced PCa skeletal metastases [24]. This functional duality — tumor-promoting in cancer cells, bone-forming in the host stroma — renders the Wnt pathway a particularly complex mediator of PCa bone metastasis. Mechanistically, Dickkopf-related protein 1 (DKK1) inhibits canonical Wnt signaling by binding to the Wnt receptors low-density lipoprotein receptor-related protein 5/6 (LRP5/6) [25]. Kremen 1 and Kremen 2 (KRM1, KRM2) bind DKK1 and cooperate in Wnt inhibition; KRM2-mediated internalization of the DKK1–LRP6 complex has been demonstrated experimentally [26]. Human genetic studies have established LRP5 as a determinant of skeletal bone mass [27], [28]. Genetic mouse models have demonstrated increased bone density following combined *Krm1*/*Krm2* disruption and a negative regulatory role of *Krm2* in bone formation [29], [30].

Human PCa cells, such as PC-3, have been shown to express high levels of DKK1 [31], [32]. DKK1 depletion reduces tumor cell growth and migration *in vitro* [31]. Following intracardiac injection, DKK1 overexpression significantly increased the number of skeletal metastatic sites in the canine Ace-1 model [33]. In a separate study using canine Probasco cells, DKK1 overexpression produced a numerical, but statistically non-significant, increase in the number of skeletal metastatic sites [34]. DKK1 also promotes subcutaneous growth of canine PCa xenografts in mice [35]. Osteoblast co-cultures and mouse calvarial organ cultures implicate *Lrp5* in PCa-induced bone formation [32]. In PC-3 cell cultures, KRM1 overexpression reduced Wnt reporter activity [36]. These findings link DKK1, LRP5, and KRM1 to PCa-associated growth, Wnt signaling, or bone remodeling; however, they do not establish how each component affects spontaneous bone metastasis *in vivo*. In a patient cohort, higher serum DKK1 was associated with poorer survival, whereas tissue expression was not prognostic [37]. Other patient tissue analyses found higher DKK1 expression in primary PCa lesions than in metastases [38], again mirroring the conflictive literature.

The available findings suggest context-dependent roles for DKK1, while direct functional *in vivo* evidence for the DKK1–LRP5/KRM1/KRM2 axis during spontaneous PCa bone metastasis remains limited. This study aimed to determine how systemic DKK1 inhibition and alterations in host Lrp5 and Krm1/Krm2 affect PCa cell dissemination and bone marrow colonization in a spontaneously metastatic human PCa xenograft model. Hypothesizing that DKK1 might play context-specific roles, we administered the humanized monoclonal antibody hDKN-01 before or after primary tumor resection. These treatment windows address different, overlapping phases of metastatic progression. Subsequent studies involved host mice carrying a heterozygous Lrp5 G170V mutation (*Lrp5*^+/G170V^) as well as homozygous *Krm1*/*Krm2* single and double knockout (KO) mice, thus distinguishing systematic DKK1 inhibition from manipulation of host receptor status.

## 2 Materials & methods

1. Cell culture

The human PCa cell line PC-3-Luc2/RGB, generated by marking PC-3 cells with firefly luciferase and RGB fluorescence (LeGO-Luc2-iCer2-Puro+, LeGO-Luc2-iV2-Puro+, and LeGO-Luc2-iC2-Puro+), was maintained as described in [8], [39], [40]. Cells were tested negative for mycoplasma infection prior to *in vivo* injection. PC-3 was selected because it reproducibly generates spontaneous, histologically verified bone marrow metastases after subcutaneous implantation on this platform [9], [10]. Unpublished evaluation of several other human PCa cell lines and PDX models under the same conditions did not yield histologically confirmable bone metastases, consistent with reports that spontaneous skeletal metastasis from a peripheral PCa primary remains difficult to reproduce in available human PCa models [6].

2. Animals

All animal experiments were approved by the local authorities (Behörde für Justiz und Verbraucherschutz, Freie und Hansestadt Hamburg, N024/2021, N021/2017, N025/2021). To explore the role of the Dkk1-Kremen/-Lrp5 axis (Suppl. Fig. S1 A) in the establishment of PCa bone metastasis *in vivo,* three independent experimental approaches were used:

2.1. Direct targeting of Dkk1

We aimed to target DKK1 during the early (pre-OP) and late phase (post-OP) of PCa bone metastasis *in vivo*. Therefore, a humanized monoclonal antibody (mAb) against Dkk1 (hDKN-01, Leap Therapeutics, Cambridge, MA, USA) was administered in our spontaneous PCa bone metastasis xenograft mouse model [9], [10]. The humanized anti-DKK1 monoclonal antibody hDKN-01 (Leap Therapeutics, Cambridge, MA, USA) and the corresponding human IgG4 isotype control (Crown Bio, San Diego, CA, USA) were prepared according to the manufacturers' instructions and diluted in sterile PBS to the required concentration for *in vivo* administration. Both experimental approaches were performed in 10–11-week-old male immunocompromised NSG (NOD.Cg-*Prkdc*^scid^ *Il2rg*^tm1Wjl^/SzJ; The Jackson Laboratory, Bar Harbor, ME, USA) mice.

2.1.1. Pre-OP approach

Mice were subcutaneously (s.c.) injected with 1 × 10^6^ PC-3-Luc2/RGB cells in 100 μL supplement-free cell culture medium on day 0. From day 1 onwards, mice received an intraperitoneal injection of 5 mg/kg hDKN-01 (50 μL/10 g body weight) or IgG4 isotype control twice a week. Treatment continued until the primary tumor reached a maximum size of ∼1 cm^3^ at which point the experiment was individually terminated (Suppl. Fig. S1B).

2.1.2. Post-OP approach

Mice were s.c. injected with 1 × 10^6^ PC-3-Luc2/RGB cells as described above, and primary tumors allowed to grow to a maximum size of ∼1 cm^3^ (pre-operative period of ∼3 weeks). Established primary tumors were consequently surgically removed and treatment with hDKN-01 (5 mg/kg, i.p., twice a week) or IgG4 isotype control initiated on the day after primary tumor removal. Treatment was continued until the individual experimental endpoint (post-operative period of ∼3 weeks or relapse tumor of ∼1 cm^3^; Suppl. Fig. S1B).

In addition, signs of beginning ulceration of the mouse skin (erythema, thinning of the overlying skin, focal depilation, early crust formation) above the primary tumor (pre-OP) or relapsing tumor (post-OP) necessitated earlier intervention (*i.e.*, primary tumor surgery) or termination of the experiment, respectively.

2.2. Targeting the Dkk1 (co)receptor axis

To explore whether targeting the Dkk1 (co)receptors affects the establishment of PCa bone metastases, mice with mutated ligand-binding domain in *Lrp5* (gain of function) or with different *Krm1*/*2* status (*Lrp5*^G170V+/−^, *Krm1*^−/−^, *Krm2*^−/−^, *Krm1*/*2*^−/−^) were backcrossed onto the NSG background (NOD.Cg-Prkdcscid Il2rgtm1Wjl/SzJ; JAX #005557) [29], [41]. After final intercrossing, NSG mice with homozygous *Krm1*, *Krm2,* or *Krm1/2* KO or normal *Krm1/2* status (control) were available. For the xenograft experiments regarding *Lrp5*, NSG mice with a heterozygous *Lrp5*^G170V^ mutation and wildtype littermates were used.

Mice were s.c. injected with 1 × 10^6^ PC-3-Luc-2/RGB cells. In one experimental approach, mice were individually sacrificed once the primary tumors had reached a size of ∼1cm^3^. In the second approach, the primary tumors were surgically resected at a size of ∼1cm^3^ and the animals were post-surgically individually followed up for ∼3 weeks or until the local tumor relapse reached a size of ∼1cm^3^ (Suppl. Fig. S1 C).

3. Bioluminescence imaging

Bioluminescence imaging (BLI) was performed as previously described. Briefly, animals were anesthetized with isoflurane and injected intraperitoneally (i.p.) with 150 mg/kg luciferin. Ten minutes after injection, mice were sacrificed, and BLI signals of all organs as well as isolated hind limbs were measured *ex vivo* using the automatic exposure setting of the IVIS 200 (Perkin Elmer, Waltham, MA, USA). Measurements were analyzed using living Image Software (Perkin Elmer) or Aura Software (Version 4. 0.8., Spectral Instruments Imaging) and BLI signal intensity was presented as total flux (photons/s).

4. Sample collection and processing
  4.1. Enzyme-Linked Immunosorbent Assay (ELISA)

On the day of sacrifice, blood was collected by cardiac puncture, ∼250 μl per sample transferred to EDTA tubes for subsequent Alu-qPCR (see below), and the remaining volume (∼500 μl) stored in 1.5 ml Eppendorf tubes on ice. Afterwards, serum was obtained by centrifugation at 10.000 rpm for 1 min (Heraeus Pico™ 17 centrifuge). The supernatant (serum) was snap frozen in liquid nitrogen and stored at −80 °C for future analysis. Circulating levels of DKK1 were quantified using a human DKK-1 ELISA kit according to the manufacturer's instructions (Invitrogen, EHDKK1).

4.2. Histology
  4.2.1. Quantification of bone metastasis

To assess the presence and size of bone metastases by histology, the respective hind limb with higher BLI signal from each mouse was fixed in 3.7% formalin for 24–48 h and processed for anti-luciferase immunohistochemistry as described [8]. Every 20th section was examined for the presence of luciferase-positive bone metastases using a light microscope. Slides containing metastases were digitized using an AxioScan Z.1 slide scanner (Zeiss, Oberkochen, Germany). Using Zeiss imaging software (ZEN 3.2), the luciferase-positive bone metastasis area [μm^2^] and the entire bone area on the respective slide [μm^2^] were determined to calculate the bone metastasis area per bone area [%] for each slide [10].

4.2.2. Bone histomorphometry

After removal of the hind limbs, the remaining skeleton was preserved for histomorphometric analysis to determine putative systemic effects of tumor growth on bone remodeling. Briefly, trabecular bone volume, number, thickness, and space were quantified on von Kossa/van Gieson stained undecalcified sections of vertebra using the Bioquant Osteo histomorphometry system (BIOQUANT Image Analysis Corp., Nashville, TN, USA).

4.3. *Alu*-qPCR

Human-specific Alu-qPCR was used as an additional measure for the metastatic load in bone marrow, blood, and organs (lung and liver). Blood was collected in EDTA tubes and stored at 4 °C until DNA extraction (within one week after harvest) and further analysis. Lung pieces were stored snap-frozen in liquid nitrogen and stored at −80 °C. Bone marrow cells were isolated from the hind limb with the lower BLI signal by centrifugation at 10.000 rpm for 1 min. The resulting bone marrow fraction was re-suspended in 0.9% NaCl and stored at 4 °C. DNA was isolated using the QIAmp DNA Blood Mini Kit or the QIAmp Mini Kit (within one week after harvest). *Alu*-qPCR was performed in a Roche Light Cycler 480 using the primers described in [10], [42]. The number of tumor cells per 60 ng of tissue DNA was calculated using a standard curve consisting of DNA ranging from 1 × 10^4^ down to 0 PC-3-Luc2/RGB cells per ml, which had been mixed with the corresponding background tissue from tumor-free mice.

5. Statistical analysis

Statistical analysis of the data was performed using GraphPad Prism Software 9 (GraphPad Software Inc., San Diego, CA, USA). Data were tested for normality distribution using the Shapiro-Wilk test. Student's *t*-test or nonparametric Mann-Whitney test was used for the comparison of two experimental groups, whereas ordinary One-Way ANOVA or Kruskal-Wallis tests were used when comparing more than two groups, with *p ≤ 0.05; **p ≤ 0.01; ***p ≤ 0.001.

## 3 Results

Prostate cancer xenograft tumor growth and distant metastasis under hDKN-01 treatment.

To investigate the role of DKK1 for s.c. PCa xenograft tumor growth and spontaneous distant metastasis in NSG mice, two settings were chosen: in the Pre-OP approach, hDKN-01 was administered to the mice starting on d1 after tumor cell transplantation until the primary tumors reached a volume of approximately 1 cm^3^; at that point, the mice were euthanized to quantify distant metastases. In the Post-OP approach, tumors that had grown to 1 cm^3^ (without therapy) were surgically removed; the day after, treatment with hDKN-01 was initiated and continued for 3 weeks or until the recurrent tumors reached approximately 1 cm^3^ (both were humane endpoints of the Post-OP group).

Importantly, in both experimental settings the treatment with hDKN-01 significantly reduced the DKK1 levels in the serum of mice compared to mice receiving the IgG4 isotype control (Fig. 1A). In vertebrae, this treatment significantly increased the trabecular bone volume and number, accompanied by a decreased trabecular space whereas the trabecular thickness was not affected (Fig. 1B). For the bone histomorphometric analysis, mice from the Post-OP approach were used.

**Fig. 1:**
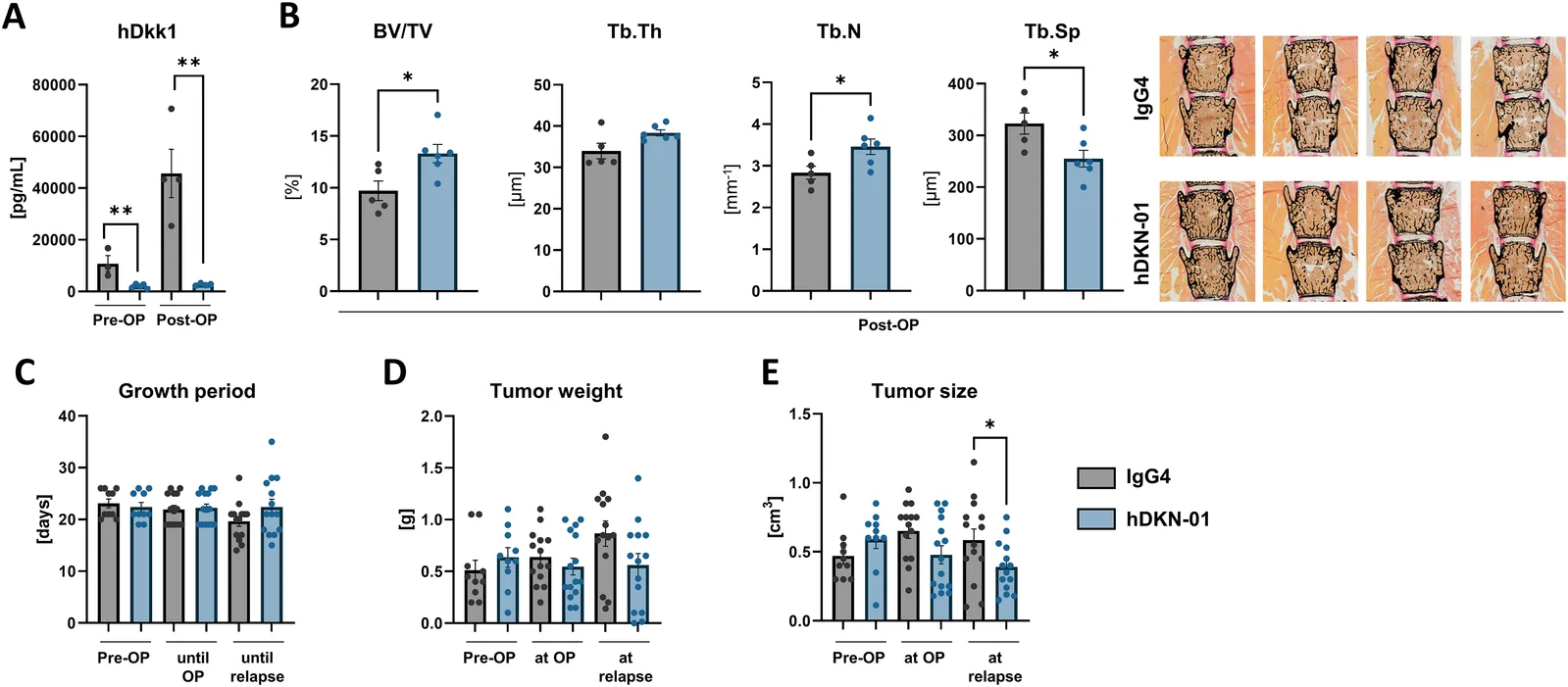
**Effects of hDKN-01 administration on bone remodeling and primary tumor growth.** DKK1 serum levels at necropsy after treatment of mice with hDKN-01 or IgG4 control **(A)**. Bone histomorphometry of non-decalcified vertebrae (von Kossa/ van Gieson stainings as illustrated) from previously tumor-bearing, post-operative, hDKN-01- or IgG4-treated mice **(B)**. Xenograft primary and post-operative relapse tumor growth parameters in mice treated with hDKN-01 or IgG4 control **(C-E)**. BV/TV - bone volume per tissue volume; Tb.Th - trabecular thickness; Tb.N - trabecular number; Tb.Sp - trabecular space. *p < 0.05, **p < 0.001.

Despite these systemic effects of hDKN-01, we observed no significant difference in primary tumor growth (Fig. 1C-E). The primary tumor growth periods, tumor weights and tumor sizes in the mice that were randomly assigned to the OP group were also similar (Fig. 1C-E). Following surgery, the treatment with hDKN-01 was started and administered until one of the two humane endpoints was reached. We observed a slight but significant reduction in relapse tumor size in the hDKN-01 group, whereas the growth period and weight of the relapse tumors remained unaffected (Fig. 1C-E).

We next assessed whether hDKN-01 treatment reduces spontaneous systemic dissemination and metastasis. In both experimental setups, the amount of human DNA per 60 ng mouse blood DNA – which provides insight into the overall hematogenous spread observed at autopsy – did not differ between the treatment groups either before or after surgery (Fig. 2A). Similarly, no difference was observed in the development of lung metastases, as assessed by BLI (Fig. 2B) and *Alu*-qPCR (Fig. 2C). Furthermore, no effect on the presence of liver metastases was observed (Fig. 2D). With regards to the establishment of bone metastases, we first performed BLI and *Alu*-qPCR, but both methods did not reveal differences in the bone metastasis loads between the groups, irrespective of the experimental setup (Fig. 2E+F). As shown in Fig. 2E+F, the BLI signal intensity as well as the amount of human DNA per 60 ng bone marrow DNA were higher in the post-OP setting when compared to the pre-OP setting (BLI: 2.65-fold, *Alu*-qPCR: 10.36-fold; considering both the IgG4 and hDKN-01 group). Therefore, we decided to focus our histological analysis on the post-OP setting. We could detect bone metastases in the hind limbs of three out of 14 mice treated with isotype control and three out of 13 mice in the hDKN-01 group. No significant difference in the bone metastasis area per bone tissue area could be determined (Fig. 2G).

**Fig. 2:**
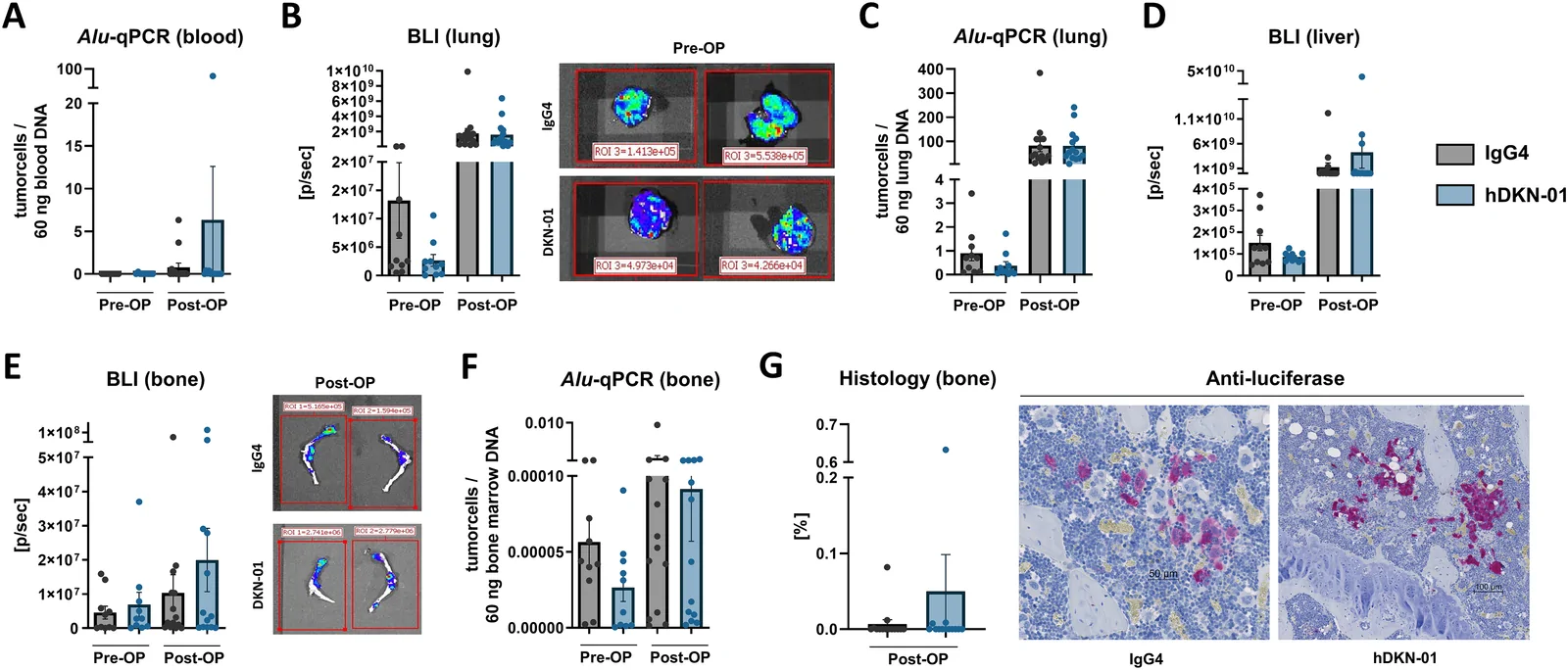
**Effects of hDKN-01 treatment on spontaneous distant metastasis.** Human DNA load in blood at necropsy determined by *Alu*-qPCR as a measure for systemic spread **(A)**. *Ex vivo* bioluminescence imaging (BLI) signals and corresponding *Alu*-qPCR of several organs quantifying distant metastasis loads in lung **(B, C)**, liver **(D),** and bone (hind limbs) **(E** **+** **F)**. Histological quantification of bone metastases (bone metastasis area per bone tissue area) based on anti-luciferase immunohistochemistry performed on every twentieth section of a 4-μm section series of the entire bone marrow cavity per bone. For the histological analysis, the hind limb with the relatively higher *ex vivo* BLI-signal was chosen, while the hind limb with a lower BLI-signal was used for *Alu*-qPCR. Representative histomorphology of marrow-localized, early metastatic lesions is shown in the embedded images **(G)**.

Prostate cancer xenograft tumor growth and distant metastasis in Lrp5-mutated mice

To explore the role of host Dkk-1 receptors for bone metastasis formation, we employed our spontaneous PCa metastasis model in NSG mice with a heterozygous gain-of-function mutation in the canonical Dkk-1 receptor Lrp5 (*Lrp5*^+/G170V^). Immunocompetent Lrp5^+/G170V^ mice have previously been described to have an altered, high bone mass phenotype [41], [43], consistent with the role of Lrp5 signaling in bone mass regulation in humans [28]. This observation could also be confirmed after backcrossing on the NSG background and after s.c. PCa xenograft tumor growth by means of increased trabecular thickness (Fig. 3A). However, there was no difference in the primary or relapse tumor growth periods, tumor weights, or sizes between *Lrp5*^+/G170V^ and wildtype (NSG) littermate controls (Fig. 3B-D).

**Fig. 3:**
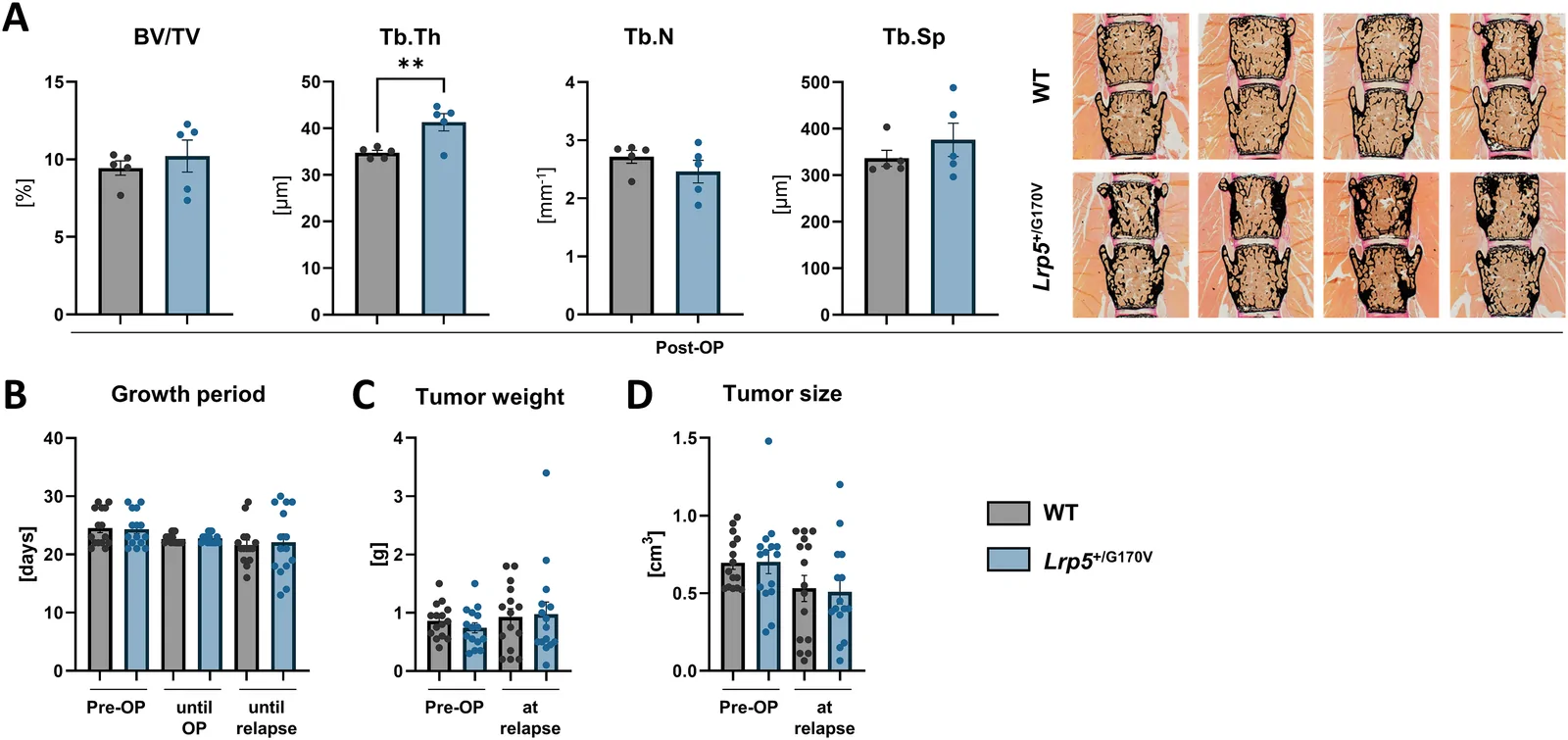
**Bone formation and primary tumor growth in *Lrp5***^**+/G170V**^**-mutated mice.** Bone histomorphometry of non-decalcified vertebrae (von Kossa/ van Gieson stainings as illustrated) from previously tumor-bearing, post-operative *Lrp5*^+/G170V^-mutated *vs.* WT NSG mice **(A)**. Xenograft primary and post-operative relapse tumor growth characteristics in *Lrp5*^+/G170V^-mutated *vs.* WT NSG mice **(B—D)**. BV/TV - bone volume per tissue volume; Tb.Th - trabecular thickness; Tb.N - trabecular number; Tb.Sp - trabecular space. **p < 0.001.

Similarly, there was no difference in the amount of human DNA per 60 ng mouse blood DNA among the two genotypes as determined by *Alu*-qPCR in both experimental approaches (Fig. 4A). The presence of lung and liver metastases also did not change in *Lrp5*^+/G170V^ mice, irrespective of whether mice were sacrificed once the primary tumor reached a size of 1 cm^3^ or ∼ 3 weeks later after primary tumor resection (Fig. 4B-D). Finally, no difference in bone metastatic burden could be determined upon heterozygous *Lrp5* mutation, either by BLI or by *Alu*-qPCR (Fig. 4E+F). The bioluminescence signal intensity as well as the amount of human DNA per 60 ng bone marrow DNA was 3.52-fold (BLI) and 52.59-fold (*Alu*-qPCR) higher in the Post-OP setting (Fig. 4E+F). Therefore, the histological analysis of putative bone metastases was again carried out with the hind limbs of mice from the Post-OP group. Bone metastases could be found in two out of 15 wildtype (NSG) littermates and in three out of 15 *Lrp5*^+/G170V^ mice. The established metastases did not differ in size between the two groups (Fig. 4G).

**Fig. 4:**
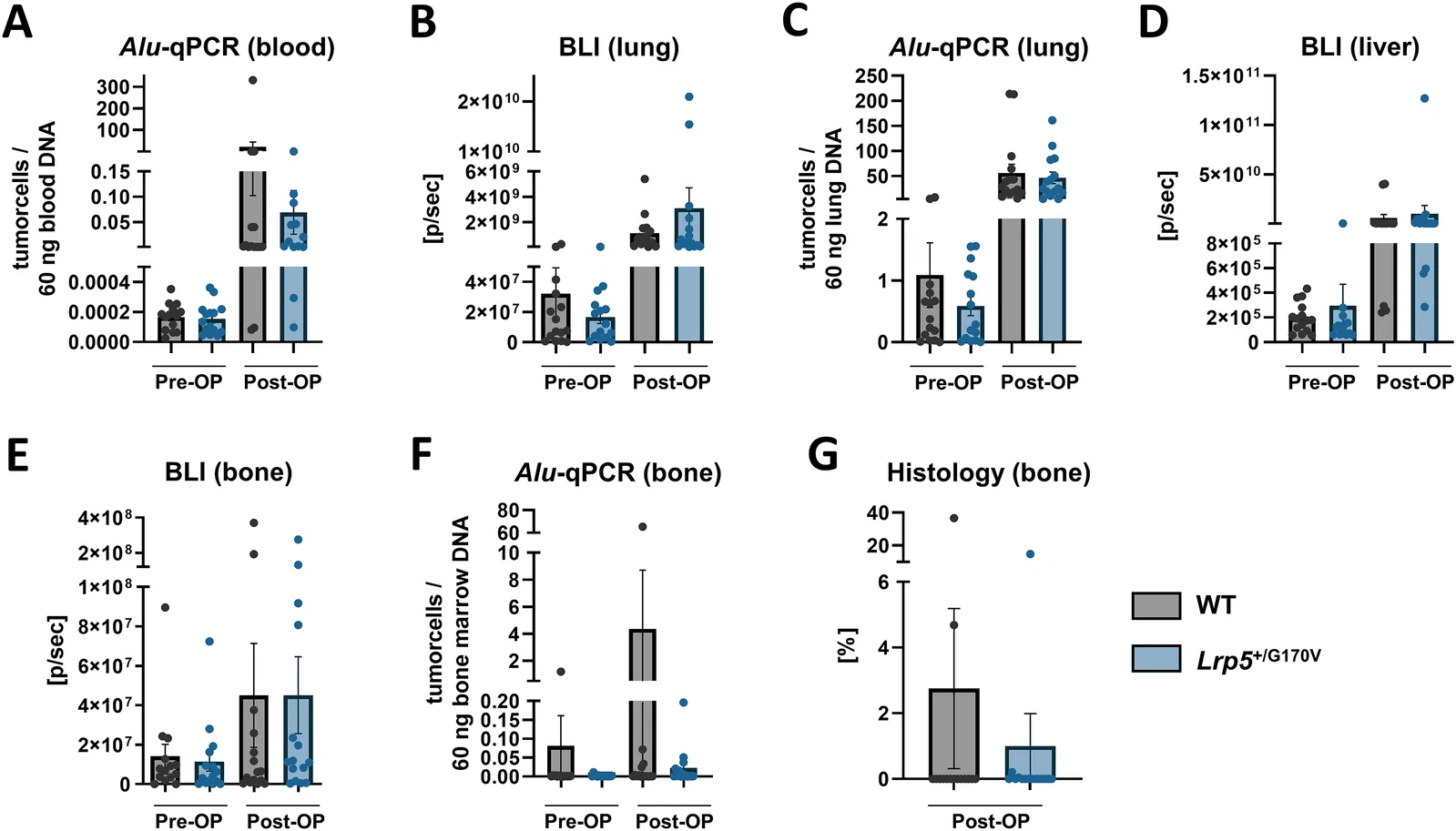
**Distant metastasis formation in *Lrp5***^**+/G170V**^**-mutated mice.** Human DNA load in blood at necropsy determined by *Alu*-qPCR as a measure for systemic spread **(A)**. *Ex vivo* BLI intensities and corresponding *Alu*-qPCR results from several organs quantifying distant metastasis loads in lung **(B** **+** **C)**, liver **(D)**, and bone (hind limbs) **(E** **+** **F)**. Histological quantification of bone metastases (bone metastasis area per bone tissue area) based on anti-luciferase immunohistochemistry **(G)**.

Prostate cancer xenograft tumor growth and distant metastasis in Krm1/2 single and double KO mice

In the next step, we investigated the capacity of PCa cells to form spontaneous bone metastases in NSG mice with different Krm1/2 (Dkk1 co-receptor) status. Compared to wildtype (NSG) control, we observed an increased bone volume, trabecular number, and decreased trabecular space in the vertebral bodies of male, 12-week-old, tumor-free *Krm1*^−/−^/*Krm2*^−/−^ double KO mice (Fig. 5A). These bone histomorphometric analyses were performed in age- and sex-matched healthy, tumor-free mice and therefore reflect genotype-dependent alterations in bone remodeling independent of tumor growth. The other genotypes (*Krm1*^−/−^ or *Krm2*^−/−^ single KO) did not show significant alterations compared to WT mice, although *Krm1* single KO mice to some extent resembled the bone phenotype of *Krm1*/*2* double KO mice (Fig. 5A). Neither primary nor relapse tumor growth (growth period, weight, size) was affected by Krm1/2 single- or double-KO (Fig. 5B-D).

**Fig. 5:**
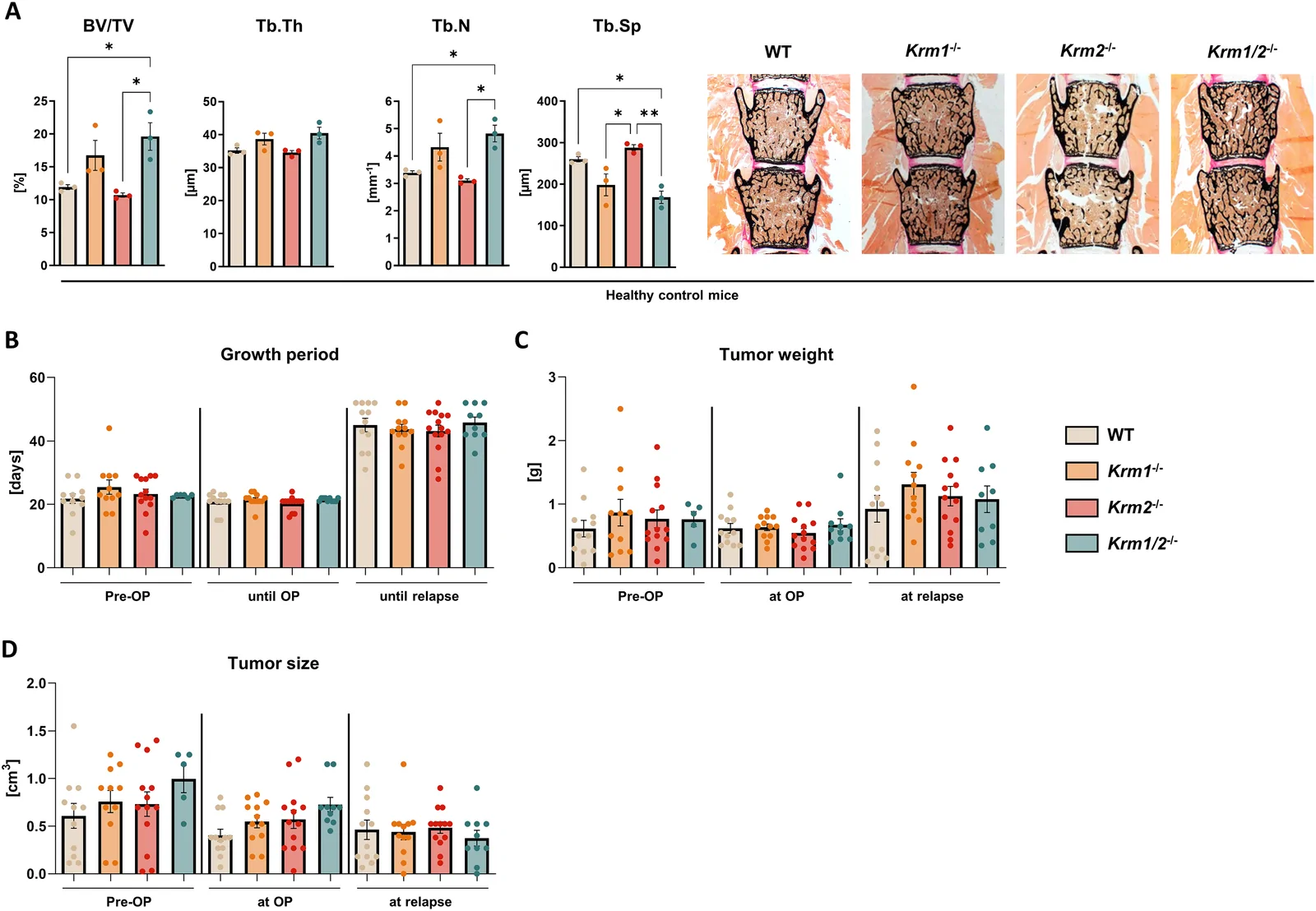
**Bone remodeling and primary tumor growth in *Krm1* and/or *Krm2* knockout mice.** Histomorphometric analysis of non-decalcified vertebral bodies (von Kossa/ van Gieson) from age- and sex-matched, healthy control *Krm1*^−/−^, *Krm2*^−/−^, *Krm1*/*2*^−/−^*vs.* WT NSG mice **(A)**. Xenograft primary and post-operative relapse tumor growth dynamics across the different genotypes **(B—D)**. BV/TV - bone volume per tissue volume; Tb.Th - trabecular thickness; Tb.N - trabecular number; Tb.Sp - trabecular space. *p < 0.05; **p < 0.001.

The analysis of human DNA in blood *via Alu*-qPCR did not reveal a difference between the genotypes in both experimental setups (Fig. 6A). Lung metastasis burden, as determined by BLI and *Alu*-qPCR, was also not altered between the genotypes (Fig. 6B+C). Likewise, BLI and *Alu*-qPCR analysis of the hind limbs did not reveal a difference in the overall bone metastasis burden (Fig. 6D+E). Importantly, however, histological analysis of the hind limbs identified DTCs without detectable early bone metastatic colonies in *Krm2*^−/−^ mice: while single DTCs could be detected in the bones of mice from all groups (WT: 2/12 mice, *Krm1*^−/−^: 2/13 mice, *Krm2*^−/−^: 3/10 mice, *Krm1*/*2*^−/−^: 6/12 mice), multi-cellular, early bone colonies could not be detected in *Krm2*^−/−^ mice (WT: 3/12 mice, *Krm1*^−/−^: 2/13 mice, *Krm2*^−/−^: 0/10 mice, *Krm1*/*2*^−/−^: 4/12 mice) (Fig. 6F). Illustrative examples of bone-metastatic lesions are shown in Fig. 6G (anti-luciferase immunohistochemistry).

**Fig. 6:**
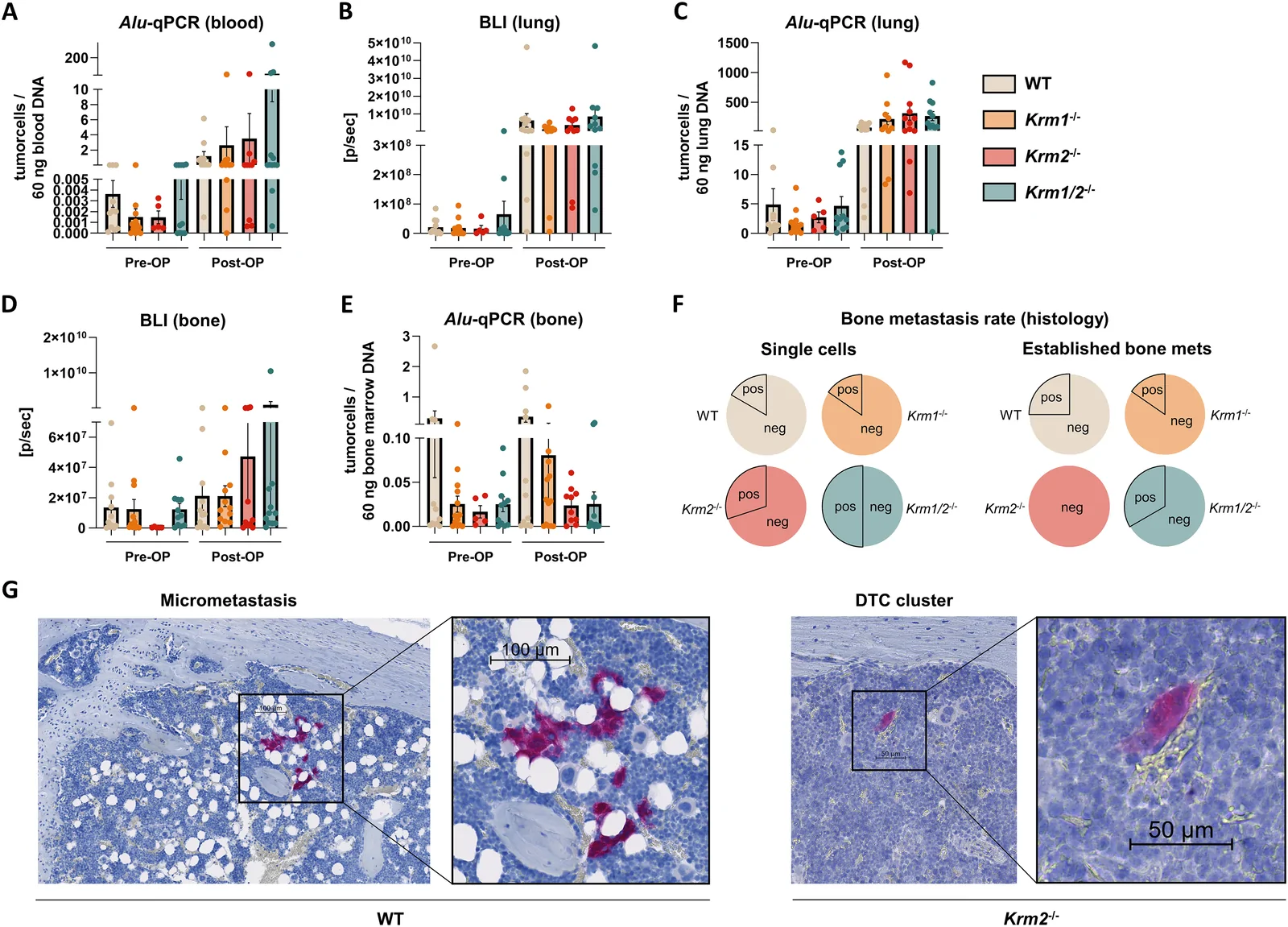
**Hematogenous metastasis formation in *Krm1* and/or *Krm2* knockout mice.** Human DNA load in blood at necropsy determined by *Alu*-qPCR to assess overall systemic dissemination **(A)**. *Ex vivo* BLI intensities and corresponding *Alu*-qPCR quantifying distant metastasis loads in lung **(B** **+** **C)** and bone **(D** **+** **E)**. Rate of animals per group showing single disseminated tumor cells (DTCs) in bone or established bone metastases across genotypes **(F)** based on anti-luciferase immunohistochemistry performed on every twentieth section of a 4-μm section series of the entire bone marrow cavity per bone. Illustrative examples of a bone micrometastasis and a DTC cluster are shown in **(G)**.

Therefore, we decided to repeat the experiment in *Krm2*^−/−^ mice (Post-OP setting only) and pooled both experiments for increased animal numbers and a more reliable readout of the histologically determined bone metastatic burden. Here, we observed a significantly increased primary tumor size and longer time to primary tumor surgery in *Krm2*^−/−^ mice when compared to wildtype (NSG) control. Primary tumor weight at the time of surgery, however, was not different between the two groups (Fig. 7A-C). The overall study duration (time from injection of tumor cells to sacrifice) was not different between the groups (Fig. 7D). With regards to the growth of relapse tumors (after surgical removal of primary tumors), we observed smaller relapse tumors in *Krm2*^−/−^ mice (volume estimated *in vivo*) but no difference in the weight of the relapse tumors (weighed at necropsy) (Fig. 7E+F). As in the preliminary experiment, no difference in circulating tumor cell DNA, lung or liver metastasis burden was observed in the expanded *Krm2*^−/−^ and WT cohorts (Fig. 7G-K). Additionally, no significant difference in bone metastatic burden was detected by *ex vivo* BLI or *Alu*-qPCR (Fig. 7L+M). However, we again observed a clear decrease in the bone metastasis burden in *Krm2*^−/−^ mice by means of histological quantification, which did not reach statistical significance (Fig. 7N).

**Fig. 7:**
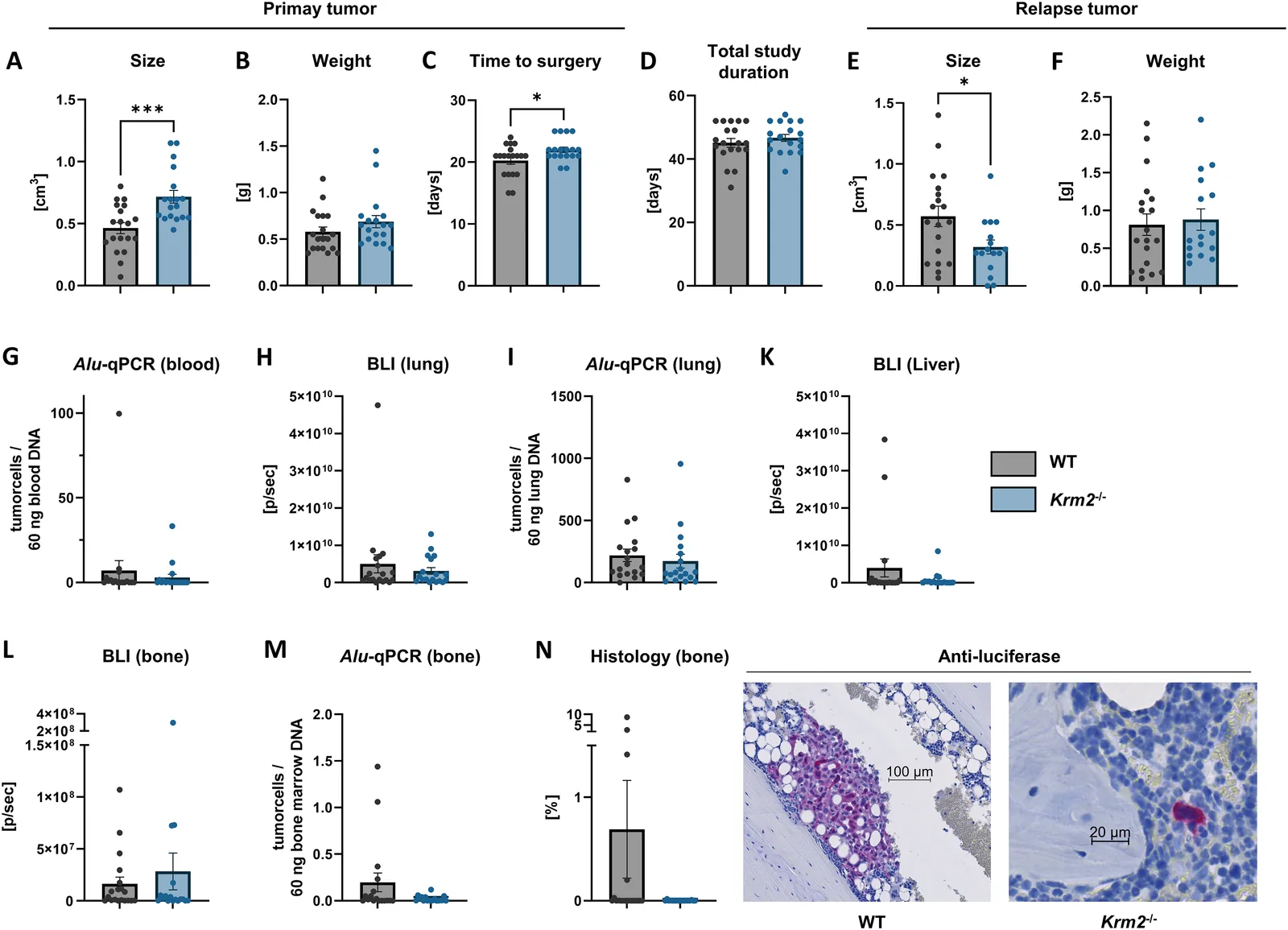
**Expansion of the bone metastasis experiment in *Krm2* knockout mice.** Given the lack of outgrowing bone metastases in *Krm2* knockout NSG mice (Fig. 6), further *Krm2*^−/−^ mice were compared to WT NSG mice, specifically in the Post-OP setting. S.c xenograft primary and relapse tumor growth characteristics **(A-F)**. Quantification of human DNA in blood **(G)** as well as distant metastasis to lung, liver, and bone at necropsy, as determined by *Alu*-qPCR and *ex vivo*-BLI **(H-M)**. Histological quantification of bone metastases (bone metastasis area per bone tissue area) based on anti-luciferase immunohistochemistry on every twentieth section of a 4-μm section series of the entire bone marrow cavity per bone **(N)**. Images embedded in **(N)** show an established bone metastasis (as found in ∼25% of WT NSG mice) and a single DTC (the only metastatic phenotype detectable in *Krm2*^−/−^ NSG mice).

To exclude primary tumor burden as a confounding variable for bone metastatic counts, endpoint tumor volumes and weights were correlated with individual bone metastatic burden. Importantly, primary tumor volume did not correlate significantly with bone metastatic burden as determined by *Alu*-qPCR (*r* = −0.022; *p* = 0.901) or histology (*r* = −0.016; *p* = 0.923). Time from tumor cell injection to primary tumor surgery likewise showed no significant correlation with *Alu*-qPCR (*r* = −0.024; *p* = 0.891) or histological metastatic burden (*r* = −0.222; *p* = 0.186). No significant correlation was observed between relapse tumor size and bone metastatic burden (*Alu*-qPCR: *r* = 0.209, *p* = 0.242; histology: *r* = 0.198, *p* = 0.255). These findings indicate that the observed differences in bone colonization between Krm2−/− and WT mice were independent of primary tumor burden.

## 4 Discussion

Despite extensive literature, the functional role of DKK1 for PCa bone metastasis remains unclear. Indeed, it has been shown that metastasis-associated gene expression is altered upon DKK1 KO in PCa cells [31] and that DKK1 significantly increased the number of skeletal metastatic sites after intracardiac injection of canine Ace-1 tumor cells in mice [33]. Clinically, higher serum DKK1 levels have been reported in patients with metastatic compared to localized PCa [44]. However, a study including several cancer types found no significant difference in serum DKK1 according to bone metastatic status [45]. In patient tissue, DKK1 protein expression was reported to increase early in PCa development and to be higher in primary PCa lesions compared to metastases, including skeletal metastases [38]. Nevertheless, DKK1 has been reported to regulate the PCa - bone cell interaction and to consequently modulate the tumor cell-induced bone remodeling in PCa bone disease [32], [34], [35], [46], [47], [48]. Bone microenvironment (BME)-derived DKK1 might also affect disseminated PCa cells that enter the bone marrow. The role of DKK1 in PCa dormancy remains to be elucidated, but it has already been described as a dormancy-inducing factor in experimental lung and breast cancer models [49].

Given this conflicting literature, we hypothesized that DKK1 might have context-specific effects on bone metastasis with a rather pro-metastatic role during the early phase of the metastatic cascade [31], [50], but a rather outgrowth-inhibiting role in the BME during the late phase of the metastatic cascade (*e.g.*, by inducing dormancy [49]). At first glance, a pro-metastatic role for DKK1 appears paradoxical, since canonical Wnt signaling is broadly associated with tumor cell invasion, EMT, and metastatic dissemination across cancer types — properties that a Wnt inhibitor such as DKK1 would be expected to suppress. However, several mechanisms could reconcile this apparent contradiction. First, DKK1 signals not only through LRP5/6 but also through the transmembrane receptor CKAP4, activating PI3K/AKT-dependent pro-migratory and pro-proliferative effects independently of Wnt pathway status [51]. Second, the pro-metastatic functions of canonical Wnt signaling are predominantly described in epithelial tumor cells, whereas in osteoblasts the same pathway drives differentiation and bone formation. DKK1-mediated Wnt inhibition in the bone stromal compartment may therefore have consequences for the metastatic niche that are mechanistically distinct from any direct effect on tumor cell Wnt signaling. Third, whereas EMT facilitates invasion and intravasation at the primary site, metastatic outgrowth at distant sites has been associated with the reverse transition — MET — which may be Wnt-dependent in some contexts [52], [53], potentially linking DKK1-mediated Wnt suppression to impaired outgrowth at the metastatic site.

Consequently, we tested whether neutralizing DKK1 with hDKN-01 decreases spontaneous systemic spread in the early phase (pre-OP approach), but increases bone metastasis outgrowth in the late phase (post-OP approach) of the metastatic process. Across several previously tested human PCa cell lines, the chosen PC-3 model was known to produce the highest amounts of spontaneous, histologically detectable bone metastases following s.c. implantation (at least in ∼15% of the tested mice) [12], [54]. In the present experiments, hDKN-01 reduced measurable serum DKK1 levels and expectedly affected bone remodeling, supporting its biological activity. However, we observed no significant effects of the treatment on bone metastasis, overall dissemination (blood), or metastasis to other sites (lung, liver). Primary and relapse tumor growth also remained largely unaltered, apart from a small but statistically significant reduction in relapse tumor volume. This is in contrast to an earlier study showing that hDKN-01 treatment does significantly inhibit primary PC-3 xenograft tumor growth *in vivo* [55]. However, in that study, the authors demonstrated that functional NK cells (as present in SCID mice) were required to achieve such an effect. Our experiments lacked functional NK cells due to the IL-2 receptor gamma chain KO in NSG mice. NSG mice were, however, necessary to generate detectable spontaneous bone metastases at least in a fraction of mice, which was the primary goal of this study. Importantly, we have previously demonstrated that the burden of spontaneous bone and lung metastases in NSG mice is approximately 10 times higher than in SCID mice [8]. To investigate the NK-cell-dependent effects of hDKN-01 on bone metastasis, it would be informative to establish a human PCa model that reliably develops spontaneous bone metastases in NK cell-competent mice following heterotopic or orthotopic primary tumor growth.

In the tested model, hDKN-01 is supposed to neutralize both DKK1 derived from human tumor cells and Dkk1 derived from the mouse BME. To further dissect putative effects of DKK1/Dkk1 on bone metastasis depending on its respective receptor binding, we subsequently examined mice with mutated *Lrp5* or KO of *Krm1*, *Krm2*, or *Krm1*/*Krm2*. While the expected alterations in bone remodeling [29] could again be confirmed in NSG mice, a numerical reduction in histologically determined bone metastatic burden was observed in *Krm2* single KO mice only. Although individual DTCs were detected in 3/10 mice in the initial *Krm2* single KO cohort, no established metastases were found in these ten mice, compared with 3/12 mice showing outgrowing metastases in the WT group. The overall low incidence of established bone metastases is a limitation of this study (13–33% of mice across all experimental groups except *Krm2* KO) and restricts the precision of the histological comparison. The reduction in histologically quantified bone metastatic burden did not reach statistical significance*.* It would therefore be interesting to analyze the role of *Krm2* for bone metastasis in a human PCa model with higher spontaneous bone metastasis potential. It may be possible to develop such a model by isolating tumor cells from spontaneous bone metastases, propagating them *in vitro*, and re-injecting them into new recipient mice. So far, it has been concluded that the efforts of other groups in this area have been limited to modeling only small parts of the overall process of bone metastasis [6]. However, after several cycles of implantation as a primary tumor and recovery from the bone, it may be possible to generate a human PCa subline with enhanced potential for spontaneous bone metastasis based on xenografts in mice.

Concurrent with the numerical reduction in histological bone metastatic burden, the *Krm2*^−/−^ mice in the expanded experiment had significantly larger primary tumors, a longer time interval to primary tumor surgery, and smaller relapse tumor volumes. As detailed in the results, however, none of these tumor growth parameters correlated significantly with bone metastatic burden by either *Alu*-qPCR or histology. The observed impairment of early bone colonization in *Krm2*^−/−^ mice was therefore most likely independent of the concurrent tumor growth alterations.

Surprisingly, the absence of established bone metastases in the *Krm2*^−/−^ cohort was not evident in *Krm1*/*Krm2* double KO mice. On the contrary, DTCs and developing metastases were actually, by trend, most common in *Krm1*/*Krm2* double KO mice, which could be related to the increased trabecular bone volume in these mice (vicious cycle, [2]). At the same time, bone metastasis was not increased in *Krm1*^−/−^ mice. This pattern suggests that the association between *Krm2* deficiency and bone metastasis may depend on *Krm1*, but does not establish a genetic rescue or a molecular interaction. The lack of a mechanistic explanation is a further major limitation of this study. Host-wide knockouts do not identify the responsible cell compartment or establish that the observed pattern depends on DKK1. Future studies should employ *e.g.*, co-incubation experiments of tumor cells with cells from the BME from *Krm1*/*2* single and double KO *vs.* WT mice with the aim to discover potential Krm1/2-dependent mechanisms as part of the complex crosstalk between tumor and BME cells.

A further limitation of the present study is that the metastatic lesions observed in the mouse model do not entirely reflect the typical histomorphology of established bone metastases in PCa patients. The marrow-restricted, non-osteoblastic morphology is compatible with early colonization but the present experiments do not establish whether these lesions would subsequently develop an osteoblastic phenotype. Isolated DTCs and micrometastases are unlikely to exhibit the terminal osteoblastic phenotype observed in advanced human disease. Additionally, PC-3 cells tend to display an osteolytic phenotype in bone metastasis models [6], a cell-line-intrinsic limitation. Therefore, our findings should not be generalized to terminal, osteoblastic-dominant disease.

As mentioned earlier, the use of the PC-3 cell line only remains a genuine limitation. Spontaneous skeletal metastasis from a peripheral PCa primary remains difficult to reproduce in available models [6]; consistent with this, our spontaneously metastatic C5 PDX model showed bone marrow tumor cell loads measured by *Alu*-PCR that declined markedly post-surgically [13], and evaluation of additional cell lines and PDX models by our laboratory under comparable conditions did not yield confirmable bone metastases. Because this study specifically targets host-dependent regulation of marrow arrest and outgrowth, substituting another line *via* intracardiac or intraosseous delivery would test a mechanistically different process rather than replicate these findings. PC-3 was therefore the most suitable model available to us for this question, though confirmation of the Krm2 phenotype in additional, independently derived models remains an important goal for future work.

In summary, our data show a marginal effect of hDKN-01 on tumor growth and no detected anti-metastatic effect in s.c. human PCa xenografts in NSG mice lacking functional NK cells. Both single DTC and establishing bone colonies were detectable at comparable, but overall low incidence in *Lrp5*-mutated as well as *Krm1*- and *Krm1*-/*Krm2*-KO mice. In contrast, *Krm2* single KO exhibited comparable DTC numbers, but showed an absence of establishing bone metastases. These findings suggest an association between host *Krm2* status and metastatic outgrowth in bone that appears to depend on *Krm1*, but do not establish a DKK1-dependent mechanism. The underlying mechanisms of this apparently very complex interplay remain to be determined.

**Supplementary Figure S1:**
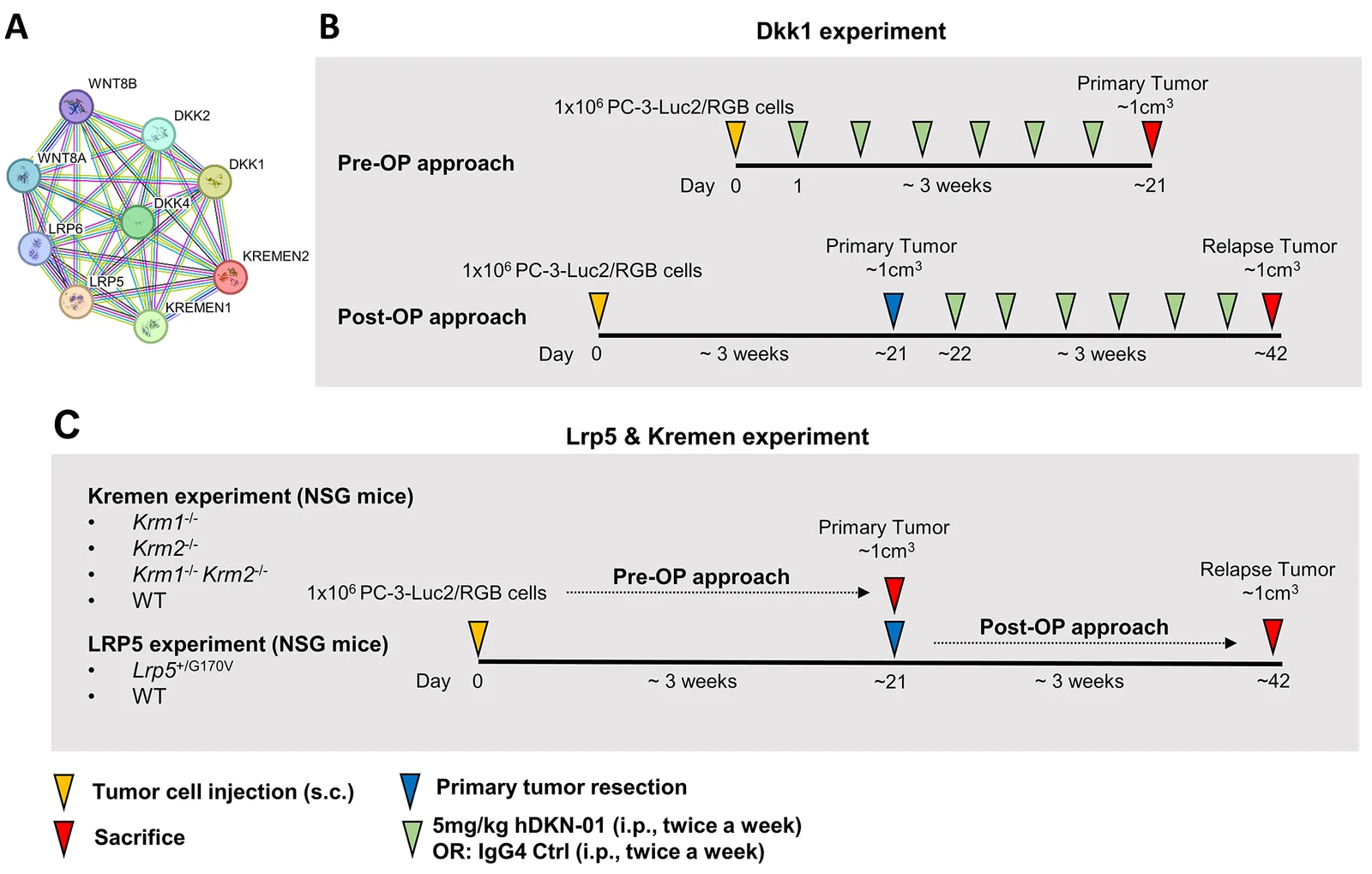
**Illustration of the study design.** Interactome representation of known protein-protein interactions between Wnt’s, Dkk’s and their Lrp and Krm receptors **(A)**. The present study analyzed the effects of hDKN-01, a clinical phase 1-2 humanized monoclonal antibody against DKK1, on subcutaneous primary tumor growth and spontaneous bone metastasis formation in a human prostate cancer xenograft model, discriminating potential effects in the early (pre-operative) from those in the late (post-operative) metastatic phase **(B)**. Likewise, tumor growth and bone metastasis were compared in mice with or without mutation of the ligand-binding domain of *Lrp5* or genetic knockout of *Krm1* and/ or *Krm2***(C)**. As a model for human PCa, PC-3 cells marked with firefly luciferase and RGB fluorescence (individual red/green/blue labeling) were used throughout the study. NSG - NOD SCID IL-2Rγ-KO mice; WT - wildtype; s.c. - subcutaneous; i.p. - intraperitoneal; Ctrl - control.

## CRediT authorship contribution statement

**By Marie-Therese Haider:** Writing – review & editing, Writing – original draft, Validation, Methodology, Investigation, Formal analysis, Data curation. **Vera Freytag:** Writing – review & editing, Validation, Methodology, Investigation, Formal analysis, Data curation. **Anke Baranowsky:** Writing – review & editing, Investigation, Formal analysis. **Farbod Bahreini:** Writing – review & editing, Visualization, Validation, Investigation. **Christof Niehrs:** Writing – review & editing, Resources. **Thorsten Schinke:** Writing – review & editing, Resources. **Michael Amling:** Writing – review & editing, Resources. **Tobias Lange:** Writing – review & editing, Writing – original draft, Supervision, Software, Resources, Project administration, Methodology, Conceptualization.

## Declaration of competing interest

The authors declare that they have no known competing financial interests or personal relationships that could have appeared to influence the work reported in this paper.
